# LncRNA FEZF1-AS1 promotes non-small lung cancer cell migration and invasion through the up-regulation of NOTCH1 by serving as a sponge of miR-34a

**DOI:** 10.1186/s12890-020-1154-6

**Published:** 2020-04-29

**Authors:** Shangxiao Huang, Chunjun Li, Jianfeng Huang, Penghui Luo, Dunchang Mo, Hanlei Wang

**Affiliations:** 1 Department of Radiotherapy, The Third Affiliated Hospital of Guangxi Medical University, No.13 Dancun Road, Nanning, Guangxi 530031 People’s Republic of China; 2 Department of Pathology, The Affiliated Tumor Hospital of Guangxi Medical University, Nanning, Guangxi 530031 People’s Republic of China

**Keywords:** FEZF1-AS1, Non-small cell lung cancer, miR-34a, NOTCH-1

## Abstract

**Background:**

The involvement of lncRNA FEZF1-AS1 has been analyzed in many types of cancers, while its roles in non-small cell lung cancer (NSCLC) remains unclear. We then explored the role of FEZF1-AS1 in NSCLC.

**Methods:**

qPCR and western blot were performed to measure gene expression. FEZF1-AS1, miR-34a, and NOTCH-1 were overexpressed to analyze the relationship between them. Transwell assays were performed to analyze the effects of transfections on cell invasion and migration.

**Results:**

FEZF1-AS1 was up-regulated in NSCLC patients. Increased expression levels of FEZF1-AS1 were observed with the increase in clinical stages. Bioinformatics analysis showed that miR-34a can bind with FEZF1-AS1. In NSCLC tissues, NOTCH-1 and FEZF1-AS1 were positively correlated. In NSCLC cells, over-expression of FEZF1-AS1 resulted in up-regulated expressions of NOTCH-1, while miR-34a over-expression mediated down-regulated expressions of NOTCH-1. In addition, FEZF1-AS1 and miR-34a did not alter each other, while bioinformatics analysis showed that miR-34a can bind FEZF1-AS1. Analysis of cell migration and invasion showed increased cell invasion and migration rates after FEZF1-AS1 and NOTCH-1 over-expression. MiR-34a played the opposite role and reduced the effects of FEZF1-AS1 over-expression.

**Conclusions:**

FEZF1-AS1 promoted NSCLC cell migration and invasion through the up-regulation of NOTCH1 by serving as a sponge of miR-34a.

## Background

For decades, lung cancer ranks the first place for both mortality and incidence among all cancers [1]. In 2018, 2,093,876 new cases of lung cancer were diagnosed and 1,761,007 deaths caused by lung cancer were recorded [1, 2]. NSCLC accounts for more than 85% of all cases [3, 4]. Moreover, most NSCLC patients are diagnosed with tumor metastasis to other parts of the body and the 5-year overall survival rate is as low as 10% [5, 6]. Therefore, novel therapeutic regimens are needed.

Smoking habit is closely correlated with the incidence of NSCLC [7], while NSCLC also affects non-smokers [8]. As another factor, genetic alterations also contribute to the occurrence and progression of NSCLC [9]. Notch homolog 1 (NOTCH-1) plays critical roles in cancer development by inducing epithelial-mesenchymal transition, promoting cell stemness and enhancing cell invasion [10, 11]. Some miRNAs, such as miR-34a, targets NOTCH-1 to inhibit cancer progression [12]. Through different ways, such as miRNA sponge or methylation regulator, lncRNAs interact with miRNAs to participate in diverse biological processes [13]. LncRNA FEZF1-AS1 has been reported to promote the progression of several cancers, such as lung cancer [14, 15], while the functionality remains to be further elucidated. In this study, we performed RNA-RNA interaction prediction using IntaRNA 2.0 and we found that miR-34a may bind FEZF1-AS1. Therefore, we will be focused to analyze the relationship between FEZF1-AS1 and miR-34a in NSCLC and the effects on NOTCH-1.

## Methods

### NSCLC patients

In this study, we selected 39 male and 27 female NSCLC patients (age: 35 to 67 year-old; mean age: 51.1 ± 5.6 year-old) from 177 cases of NSCLC admitted to The Third Affiliated Hospital of Guangxi Medical University between March 2016 and April 2019. This study was approved The Third Affiliated Hospital of Guangxi Medical University Ethics Committee. Inclusion criteria: 1) newly diagnosed NSCLC; 2) no therapies were initiated. Exclusion criteria:1) complicated with other clinical disorders; 2) recurrent NSCLC; 3) therapies were initiated; 4) previous history and/or family history of malignancies. 66 NSCLC patients were informed with the experiment design and potential publication of this paper, and all 66 patients signed informed consent. Clinical staging was performed using AJCC system. Based on clinical data, the 66 patients included 10, 12, 23 and 21 cases at stage I-IV, respectively.

### NSCLC tissue samples and cells

Fine needle aspiration was performed on all patients to collect paired NSCNC and non-tumor specimens. Following histological confirmation, all tissue samples were immediately subjected to RNA isolations and following experiments.

H1993 human NSCLC cell line (ATCC, USA) was used in this study. Cell culture medium was prepared by mixing RPMI-1640 Medium (90%) with FBS (10%). A 95% humidity and 5% CO_2_ incubator was used to cultivate cells.

### Vectors, miRNA mimic and transfections

Negative control (NC) miRNA and the mimic of miR-34a, as well as vectors (pcDNA3.1) expressing NOTCH-1 and FEZF1-AS1, were from GenePharma (Shanghai, China). Vector (1 μg) or miRNA (40 nM) was transfection into 4 × 10^6^ H1993 cells using lipofectamine 2000 (GenePharma). To perform NC experiments, H1993 cells were transfected with empty pcDNA3.1 vector or NC miRNA. To perform Control (C) experiment, cells without transfections were cultivated until the end of transfections. Cells were cultivated for further 48 h after transfections before use.

### RNA extractions

H1993 cells were harvested and counted. All tissue samples (about 0.012 g) were ground into powder in liquid nitrogen. Total RNAs in 4 × 10^5^ cells or tissue samples were extracted using Ribozol (Sigma-Aldrich, USA). To harvest all types of RNAs (such as miRNAs), RNAs were precipitated and washed using 85% ethanol.

### RT-qPCR

DNase I was used to incubate with RNA samples for 90 min at 37 °C to achieve genomic DNA removal. In order to measure the expression levels of FEZF1-AS1 and NOTCH-1 mRNA, total RNAs were reverse transcribed into cDNA and qPCRs were performed with GAPDH as internal control. Expression of miR-34a was determined using All-in-One™ miRNA qRT-PCR Detection Kit (Genecopoeia). U6 was included to sever as the endogenous control of miR-34a. Ct values of three technical replicates were normalized using 2^-ΔΔCT^ method.

### RNA interaction prediction

The interaction between miR-34a and FEZF1-AS1 was predicted using an online program named IntaRNA 2.0 (http://rna.informatik.uni-freiburg.de/IntaRNA/Input.jsp). All parameters were the default. FEZF1-AS1 was set to be the long sequence and miR-34a was set to be the short sequence.

### Western blot

At 24 h post-transfection, H1993 cells were counted and total proteins in 4 × 10^5^ cells were extracted using RIPA solution (GenePharma). Total proteins were denatured in boiling water for 5 min. Proteins were then separated by 10% SDS-PAGE gel, transferred to PVDF membranes, and PBS (5% non-fat milk) was used to block membranes for 1 h at 24 °C. After that, rabbit anti-NOTCH-1 (1: 1200, ab52301, Abcam) and GAPDH (1: 1200, ab37168, Abcam) primary antibodies were used to incubate the membranes for 18 h at 4 °C. After that, further incubation with IgG H&L (IgG) (1:1000; ab6721; Abcam) secondary antibody was performed for 2 h at 24 °C. Signal development was performed by incubating membranes with RapidStep™ ECL detection reagent (EMD Millipore) for 5 min. Signals were processed using Image J v1.47 software.

### Transwell assays

At 24 h post-transfection, H1993 cells were counted and transferred to upper Transwell chamber (4 × 10^3^ cells in 0.1 ml serum-free medium per well). Matrigel (Millipore)-coated membranes were used in invasion assay and uncoated membranes were used in migration assay. In both cases, lower chamber was filled with medium supplemented 20% FBS. Under aforementioned conditions, cells were cultivated for 12 h and 0.5% crystal violet (Sigma-Aldrich) was used to stain cell for 20 min at room temperature. An optical microscope was used to observe stained cells.

### Data analysis

Mean ± SD values of 3 biological replicates were calculated. Paired t test was used to compare paired tissues. ANOVA Tukey’s test was used to compare multiple groups. Linear regression was used for correlation analysis. Patients were first grouped in to high and low (*n* = 33) FEZF1-AS1 level groups. The cutoff value was the median FEZF1-AS1 expression level in NSCLC. Correlations between expression levels of FEZF1-AS1 and patients’ clinical data were analyzed by Chi-squared test. *p* < 0.05 was statistically significant.

## Results

### FEZF1-AS1 was up-regulated with an increase in clinical stages of NSCLC patients

**Fig. 1:**
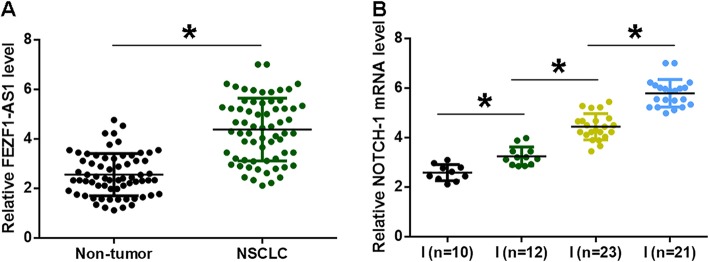
FEZF1-AS1 was up-regulated with an increase in clinical stages of NSCLC patients. QPCR and paired t-test were used to measure and compare expression levels of FEZF1-AS1 between two types of tissues (NSCLC vs. non-tumor) (**a**). ANOVA (one-way) and Tukey test were used to compare expression levels of FEZF1-AS1 in NSCLC tissues among different clinical stages (**b**). Data of 3 replicates were presented, *, *p* < 0.05

Analysis of RT-qPCR data revealed significantly higher expression levels of FEZF1-AS1 in NSCLC tissues compared to non-tumor tissues (Fig. 1a, *p* < 0.05). Expression levels of FEZF1-AS1 in NSCLC tissues among different clinical stages were compared. With the increase in clinical stages, significantly increased expression levels of FEZF1-AS1 were observed (Fig. 1b, *p* < 0.05). Chi-squared test showed that expression levels of FEZF1-AS1 in NSCLC tissues were significantly correlated with patients’ clinical stage and tumor grades, but not patients’ gender, age, subtypes and drinking and smoking habits (Table 1).

**Table 1:** Correlations between expression levels of FEZF1-AS1 in NSCLC tissues and patients’ clinical data

Items	Groups	Cases	High	Low	χ^2^	*p* value
Age	> 50(years)	35	19	16	0.55	0.46
	<=50 (years)	31	14	17		
Gender	Male	39	22	17	1.57	0.21
	Female	27	11	16		
Subtypes	Adenocarcinoma	28	14	14	0.13	0.94
	Squamous cell carcinoma	27	13	14		
	Large cell carcinoma	11	6	5		
Tumor grade	I	12	3	9	18.10	0.00042
	II	16	3	13		
	III	17	13	4		
	IV	21	14	7		
Tumor stage	I	10	2	8	0.92	0.02
	II	12	3	9		
	III	23	15	8		
	IV	21	13	8		
Smoking	Yes	41	21	20	0.06	0.80
	No	25	12	13		
Drinking	Yes	46	22	24	0.29	0.59
	No	20	11	9		

### NOTCH-1 mRNA was up-regulated and positively correlated with FEZF1-AS1

**Fig. 2:**
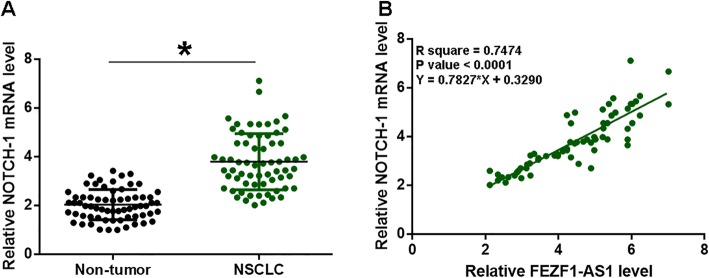
NOTCH-1 mRNA was up-regulated and positively correlated with FEZF1-AS1. QPCR and paired t-test were used to measure and compare expression levels of NOTCH-1 mRNA between two types of tissues (NSCLC vs. non-tumor) (**a**). The correlation between FEZF1-AS1 and NOTCH-1 mRNA was analyzed by linear regression (**b**). Data of 3 replicates were presented, *, *p* < 0.05

Analysis of RT-qPCR data revealed significantly higher expression levels of NOTCH-1 mRNA in NSCLC tissues compared to non-tumor tissues (Fig. 2a, *p* < 0.05). Correlation analysis showed that, across NSCLC tissues, FEZF1-AS1 and NOTCH-1 were significantly and positively correlated (Fig. 2b, *p* < 0.05).

### FEZF1-AS1 sponges miR-34a to up-regulate NOTCH-1

**Fig. 3:**
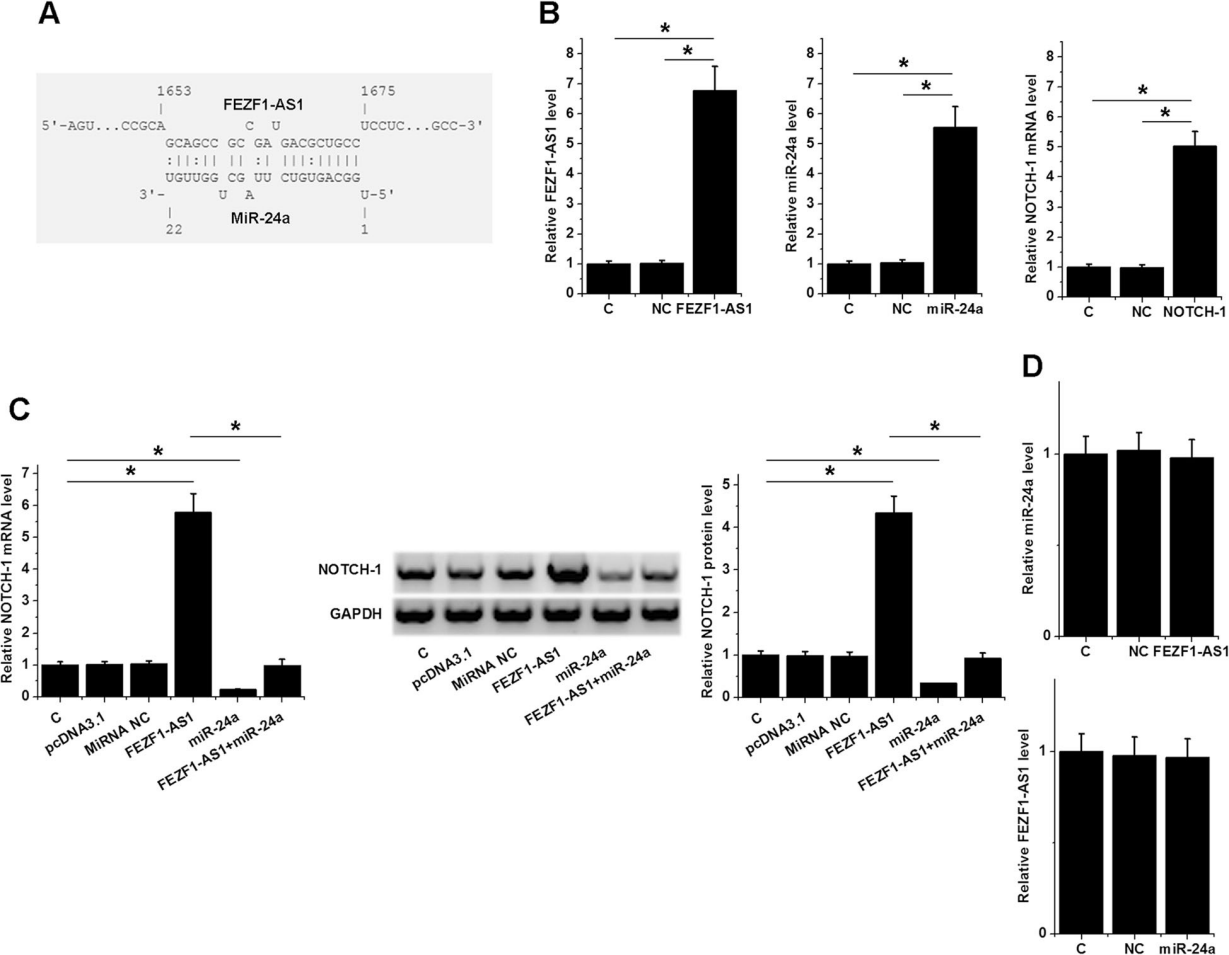
FEZF1-AS1 may sponge miR-34a to up-regulate NOTCH-1. The interaction between FEZF1-AS1 and miR-34a was predicted by IntaRNA (**a**). FEZF1-AS1 and NOTCH-1 expression vectors, as well as miR-34a mimic, were transfected into H1993 cells and the over-expression was confirmed by qPCR (**b**). The interaction between FEZF1-AS1 and miR-34a was analyzed by qPCR (**c**). The effects of FEZF1-AS1 and miR-34a over-expression on NOTCH-1 expression were analyzed by qPCR and western blot (**d**). Data of 3 replicates were presented, *, *p* < 0.05

The positive correlation between FEZF1-AS1 and NOTCH-1 mRNA indicated possible interaction between them, while miR-34a can target NOTCH-1. Therefore, the relationship between them was predicted. As shown in Fig. 3a, miR-34a can strongly bind to FEZF1-AS1. To further investigate the mechanism, FEZF1-AS1 or NOTCH-1 expression vector, or miR-34a mimic, was transfected into H1993 cells. QPCR showed that expression levels of miR-34a, FEZF1-AS1 and NOTCH-1 were significantly up-regulated compared to C and NC groups at 24 h post-transfection (Fig. 3b, *p* < 0.05). Moreover, over-expression of miR-34a and FEZF1-AS1 did not significantly alter the expression of each other (Fig. 3c, *p* > 0.05). In contrast, over-expression of FEZF1-AS1 resulted in up-regulated NOTCH-1, while miR-34a over-expression mediated down-regulated NOTCH-1 and the reduced effects of FEZF1-AS1 over-expression (Fig. 3d, *p* < 0.05). Please check Supplementary Figure 1 for representative images of Western blot results.

### FEZF1-AS1 promoted the invasion and migration of NSCLCs through the axis of NOTCH-1 and miR-34a

**Fig. 4:**
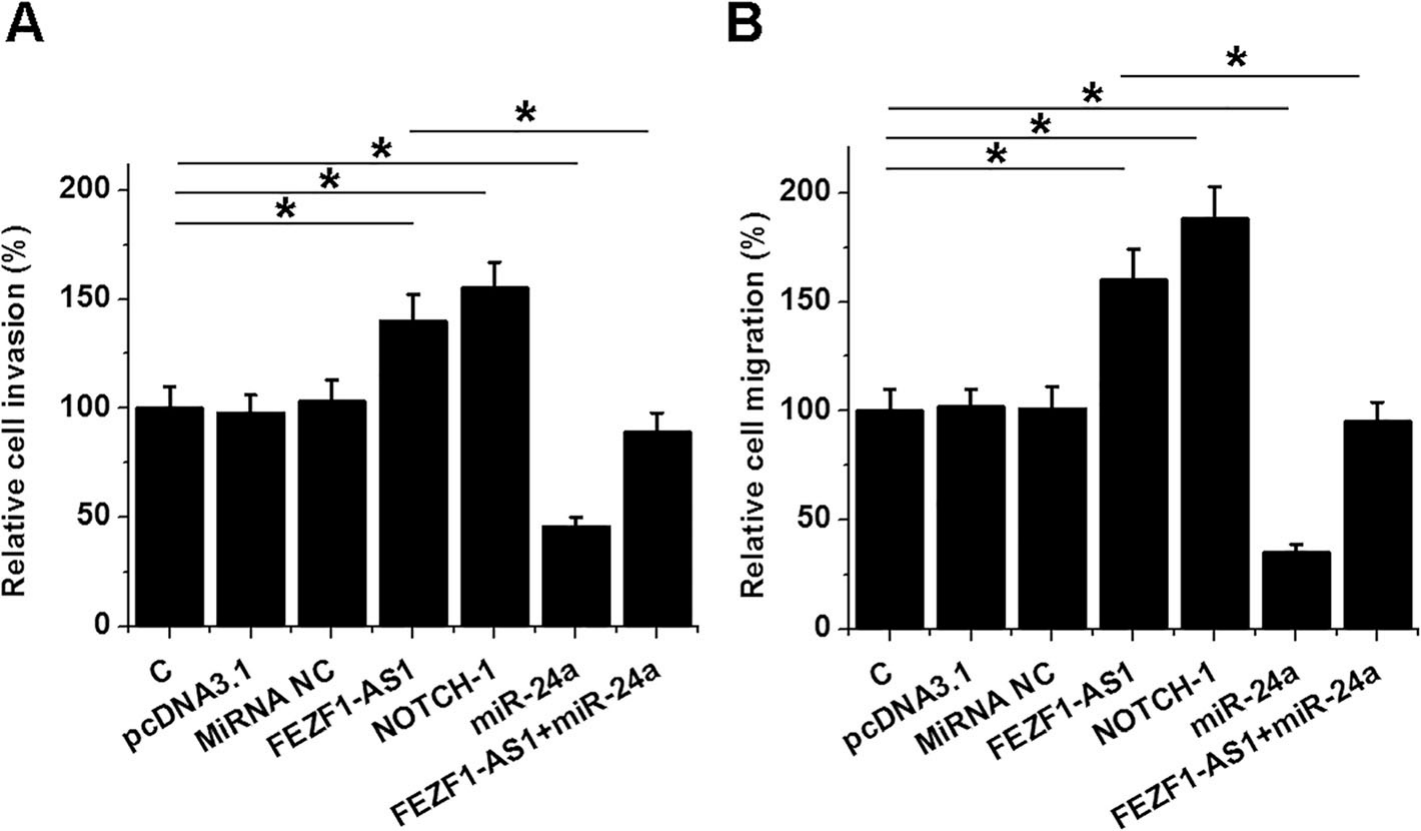
FEZF1-AS1 promoted NSCLC cell invasion and migration through miR-34a and NOTCH-1. Transwell assays were performed to analyze the effects of transfections on the invasion (**a**) and migration (**b**) of H1993 cells. Data of 3 replicates were presented, *, *p* < 0.05

Invasion (Fig. 4a) and migration (Fig. 4b) of H1993 cells in different transfection groups were analyzed by Transwell assay. Compared to C and NC groups, cells with NOTCH-1 or FEZF1-AS1 expression vector transfection exhibited significantly increased cell invasion and migration. MiR-34a played the opposite role and reduced the effects of FEZF1-AS1 over-expression (*p* < 0.05).

## Discussion

We investigated the roles of FEZF1-AS1 in NSCLC. Our study revealed that FEZF1-AS1 was up-regulated in NSCLC. In addition, FEZF1-AS1 may sever as an endogenous sponge of miR-34a to up-regulate NOTCH-1 in NSCLC cells, thereby increasing the invasion and migration of cancer cells.

The function of FEZF1-AS1 has been investigated in many types of cancers. For instance, FEZF1-AS1 is up-regulated in colorectal cancer and can regulate PKM2 signaling to promote cancer cell metastasis and proliferation [14]. In multiple myeloma, FEZF1-AS1 is also over-expressed and over-expression of FEZF1-AS1 mediates the growth of tumor [16]. In a recent study, He et al. found that FEZF1-AS1 was over-expressed and is correlated with epithelial-mesenchymal transition [15]. Consistently, this study also reported the up-regulation of FEZF1-AS1 in NSCLC, and the over-expression of FEZF1-AS1 resulted in the increased invasion and migration rates of NSCLC cells. Our study further the role of FEZF1-AS1 as an oncogenic lncRNA in NSCLC.

This study predicted the potential interactions between FEZF1-AS1 and miR-34a. It has been well established that miRNAs can regulate gene expression by direct cleavage or translation termination [17]. However, over-expression experiments showed that miR-34a has no regulatory roles in the expression of FEZF1-AS1. Therefore, miR-34a is not likely to target FEZF1-AS1. Recent studies have showed that lncRNAs may mimic the targets of miRNAs, thereby sponge miRNAs to attenuate their effects on silencing downstream genes. It is known that miR-34a can target NOTCH-1 in glioblastoma to suppress cancer growth. In this study we also observed the down-regulated NOTCH-1 at both mRNA and protein levels. Therefore, miR-34a may also target NOTCH1 in NSCLC. In addition, FEZF1-AS1 over-expression resulted in the up-regulated NOTCH-1 at both mRNA and protein levels. Therefore, our data support the speculation that FEZF1-AS1 may sponge miR-34a to up-regulate NOTCH-1, thereby promoting the invasion and migration of cancer cells. However, the mechanism remains to be further explored.

Our data may suggest FEZF1-AS1 as a potential therapeutic target for NSCLC. However, our study did not include clinical trials. Therefore, future studies are needed to perform clinical studies or animal model studies to further confirm our conclusions.

## Conclusion

In conclusion, our study confirmed the oncogenic role of FEZF1-AS1 in NSCLC and we proposed that FEZF1-AS1 may sponge miR-34a to up-regulate NOTCH-1 to promote cancer cell invasion and migration.

## Supplementary information

**Additional file 1: Figure S1.** Representative images of Western blot results. Original images here are of a biological replicate of Western blot. Data here is the same information as shown in Fig.4c.

## Data Availability

The analyzed data sets generated during the study are available from the corresponding author on reasonable request.

## Abbreviation

NSCLC: Non-small cell lung cancer
lncRNAs: Long (> 200 nt) non-coding RNAs
NOTCH-1: Notch homolog 1
