# Supplementary figures and images for: LncRNA FEZF1-AS1 promotes non-small lung cancer cell migration and invasion through the up-regulation of NOTCH1 by serving as a sponge of miR-34a
Source: BMC Pulm Med. 2020 Apr 29;20:110. doi: 10.1186/s12890-020-1154-6 (PMC7191745; doi:10.1186/s12890-020-1154-6)

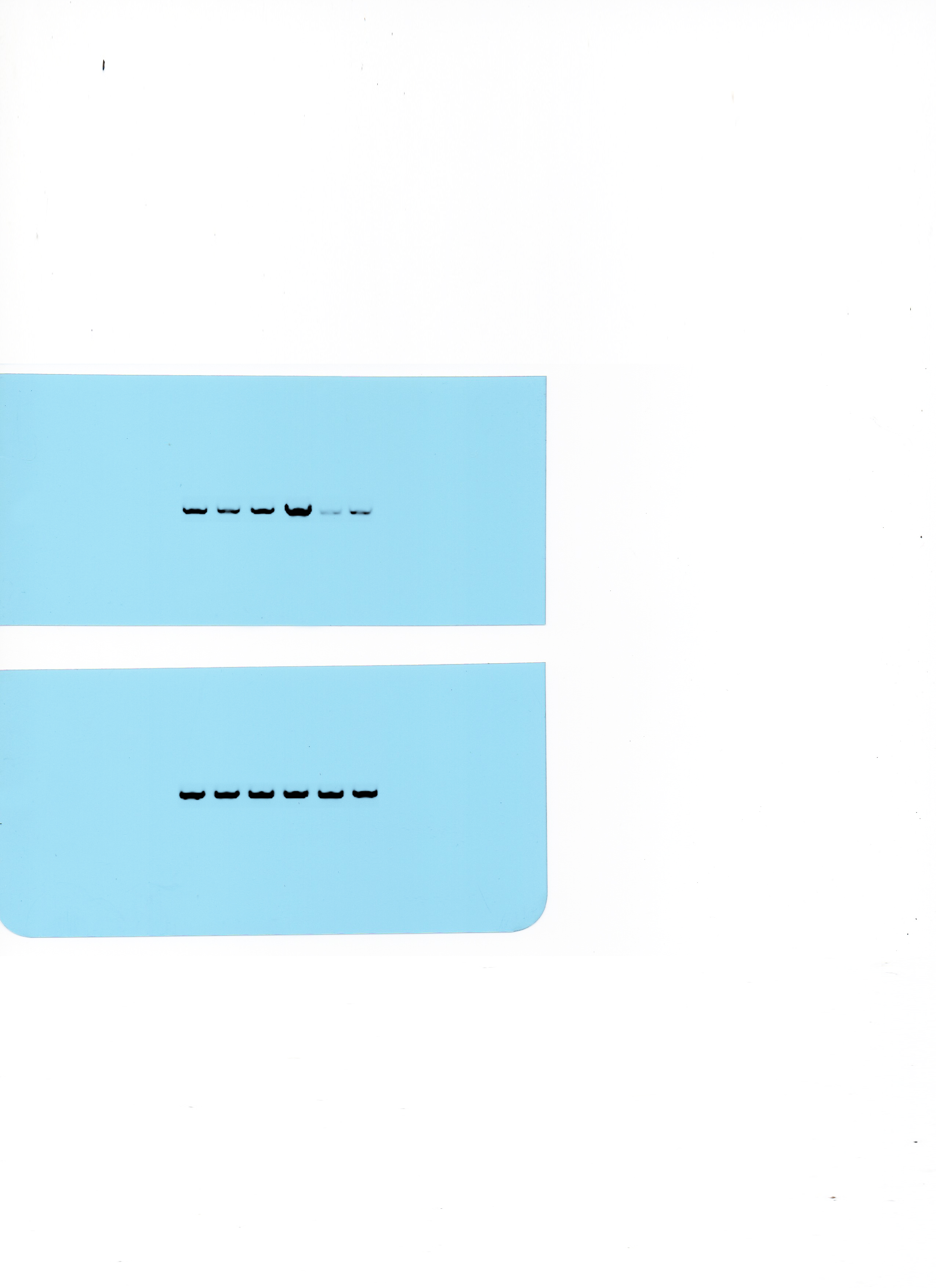

Supplement: Supplementary file 1 — Additional file 1: Figure S1. Representative images of Western blot results. Original images here are of a biological replicate of Western blot. Data here is the same information as shown in Fig.4c. [file 12890_2020_1154_MOESM1_ESM.jpg]
